# Cooperativity in Proteasome Core Particle Maturation

**DOI:** 10.1016/j.isci.2020.101090

**Published:** 2020-04-22

**Authors:** Anjana Suppahia, Pushpa Itagi, Alicia Burris, Faith Mi Ge Kim, Alexander Vontz, Anupama Kante, Seonghoon Kim, Wonpil Im, Eric J. Deeds, Jeroen Roelofs

**Affiliations:** 1 Department of Biochemistry and Molecular Biology, University of Kansas Medical Center, 3901 Rainbow Boulevard, Kansas City, KS 66160, USA; 2 Molecular, Cellular, and Developmental Biology Program, Division of Biology, Kansas State University, 338 Ackert Hall, Manhattan, KS 66506, USA; 3 Center for Computational Biology, University of Kansas, 2030 Becker Drive, Lawrence, KS 66047, USA; 4 Institute for Quantitative and Computational Biosciences, University of California Los Angeles, Los Angeles, CA 99024, USA; 5 Department of Molecular Biosciences, University of Kansas, Lawrence, KS 66047, USA; 6 Department of Biological Sciences, Lehigh University, Bethlehem, PA 18105, USA; 7 Department of Bioengineering, Lehigh University, Bethlehem, PA 18105, USA; 8 Department of Chemistry, Lehigh University, Bethlehem, PA 18105, USA; 9 Department of Integrative Biology and Physiology, University of California Los Angeles, Los Angeles, CA 99024, USA

**Keywords:** Biological Sciences, Biochemistry, Microbiology

## Abstract

Proteasomes are multi-subunit protease complexes found in all domains of life. The maturation of the core particle (CP), which harbors the active sites, involves dimerization of two half CPs (HPs) and an autocatalytic cleavage that removes β propeptides. How these steps are regulated remains poorly understood. Here, we used the *Rhodococcus erythropolis* CP to dissect this process *in vitro*. Our data show that propeptides regulate the dimerization of HPs through flexible loops we identified. Furthermore, N-terminal truncations of the propeptides accelerated HP dimerization and decelerated CP auto-activation. We identified cooperativity in autocatalysis and found that the propeptide can be partially cleaved by adjacent active sites, potentially aiding an otherwise strictly autocatalytic mechanism. We propose that cross-processing during bacterial CP maturation is the underlying mechanism leading to the observed cooperativity of activation. Our work suggests that the bacterial β propeptide plays an unexpected and complex role in regulating dimerization and autocatalytic activation.

## Highlights

• A flexible region in β propeptide regulates proteasome core particle (CP) formation
• Identified pre-holocomplex distinguishes dimerization from autocatalytic activation
• Cooperativity found in proteasome core particle (CP) activation
• Bacterial CP displayed propeptide cross-cleavage

## Graphical Abstract

**Figure:**
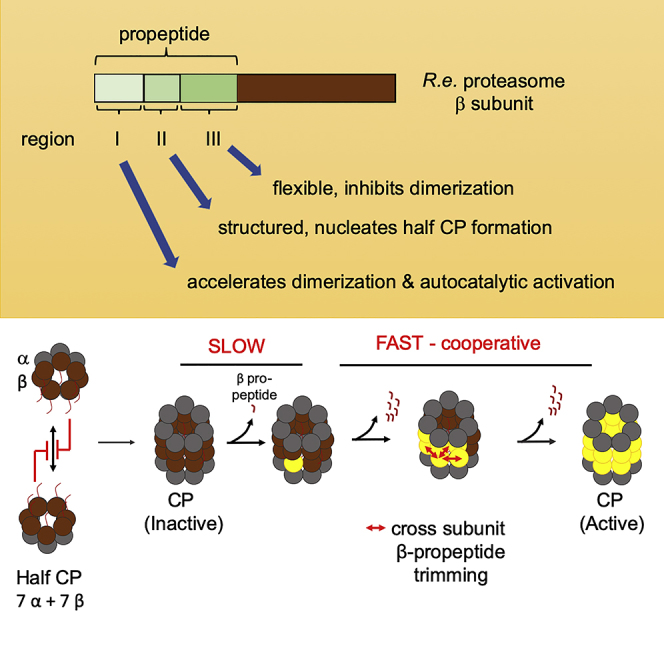

## Introduction

Protein degradation is an essential cellular process required to maintain homeostasis and to allow the cell to react efficiently to changing environmental conditions. The proteasome, one of the major proteases, is found ubiquitously in eukaryotes, archaea, and some bacterial orders like Actinomycetales and Nitrospirales (reviewed in Becker and Darwin, 2017). At its center is the structurally conserved core particle (CP) complex that consists of four heptameric rings stacked to form a hollow, cylindrical protease complex. In eukaryotes, the rings consist of seven distinct α and β subunits. However, archaeal and eubacterial genomes mostly encode only one paralog of the α and β subunit each and thus have homo-heptameric α and β rings (with an overall α_7_ β_7_ α_7_ β_7_ stoichiometry) (Becker and Darwin, 2017).

The formation of CP involves the dimerization of two half CPs (the α_7_ β_7_ “Half Proteasome", here referred to as HP), and two distinct assembly pathways for the HP have been described. One, common in eukaryotes and archaea, starts with the formation of an α subunit ring. This ring then serves as a docking site for the β subunits (Hirano et al., 2008, Zwickl et al., 1994). The second, found mainly in bacteria, starts with dimerization of α and β subunits, which then rapidly combine to form HP (Zühl et al., 1997a, Zühl et al., 1997b). The stability of the α-α versus α-β interactions, a feature that correlates with the buried surface area in the interaction, seems to be the distinguishing factor for these pathways (Hu et al., 2006, Kwon et al., 2004, Panfair et al., 2015, Zühl et al., 1997a, Zühl et al., 1997b). Regardless of the pathway, the catalytically active β subunits are synthesized in an inactive form, with an N-terminal propeptide sequence. Dimerization of the HP coincides with a proteolytic processing of some (eukaryotes) or all (archaea and bacteria) of the β subunits to form a proteolytically active CP (Becker and Darwin, 2017, Budenholzer et al., 2017, Kunjappu and Hochstrasser, 2014, Sharon et al., 2007).

The assembly process in eukaryotes is more complex since the eukaryotic CP consists of fourteen different polypeptides. Perhaps owing to this increased complexity, eukaryotic CP assembly involves at least five CP-dedicated chaperones. Some of these chaperones have orthologs in archaea, whereas none have been identified in bacteria, and prokaryote-derived CPs have been reconstituted without the need for chaperones (Kusmierczyk et al., 2011, Sharon et al., 2007, Zühl et al., 1997a). Apart from separate chaperone molecules, the propeptides of β subunits have been shown to function as “intrinsic chaperones.” For example, in yeast, the propeptide of Doa3 (i.e., β5) is required for its incorporation into the CP (Chen and Hochstrasser, 1996). Similarly, propeptides in actinobacteria are also essential for their proper folding and incorporation into higher-order complexes during assembly (Zühl et al., 1997a). The bacterial propeptides bind to the α subunits and assist in the formation of αβ heterodimers during HP assembly. The lack of dedicated chaperones in bacteria suggest that parallels between the functions of eukaryotic chaperones and bacterial propeptides exist. For example, the eukaryotic chaperone Ump1 (a.k.a. POMP in humans) is an intrinsically disordered protein that remains associated with immature HPs, preventing premature dimerization (Ramos et al., 1998). This chaperone is degraded along with other propeptides as the first substrate of an assembled and active CP (Ramos et al., 1998). This is in many ways similar to the bacterial propeptide, which is partially disordered, regulates dimerization, and is cleaved during CP maturation (Kwon et al., 2004, Zühl et al., 1997a).

A better understanding of bacterial propeptides will thus help to elucidate several fundamental evolutionarily conserved aspects of CP assembly and reveal the critical core functions of the propeptides themselves. Moreover, this may also reveal differences in the assembly process that could be exploited in the development of drugs. For example, specifically targeting the *M*. *tuberculosis* CP is thought to be of therapeutic value (Lin et al., 2009, Totaro et al., 2017). This actinomycete bacterium is the causative agent of tuberculosis (Tb), a major disease with ∼9 million new cases each year and about 1.5 million deaths (Zumla et al., 2015). Furthermore, recent data suggest that anti-PD-1 drugs used and tested against a variety of cancers are associated with higher abundance of Tb (Barber et al., 2019). In *M*. *tuberculosis*, the prokaryotic ubiquitin-like protein (Pup)-proteasome system, a bacterial conjugating system with parallels to the Ubiquitin-Proteasome System of eukaryotes, is important for virulence of this pathogen (Cerda-Maira et al., 2010, Darwin et al., 2003, Gandotra et al., 2007). Thus, a mechanistic understanding of the function of bacterial CP assembly and the role of propeptides might reveal ways how we can interfere with CP assembly, which could be developed in therapeutic targets for Tb treatment.

In this work, we used a bioinformatics approach to define three distinct regions in the bacterial *β* subunit propeptides based on their conservation patterns. Using *in vitro* reconstitutions and molecular dynamics studies, we identified a role for the N-terminal region of the propeptide in regulating the speed of HP dimerization and the autocatalytic activation of the CP. Based on these data, we propose a mechanism for the activation of the CP that involves cooperativity in the processing of propeptides between β subunits present in CP.

## Results

### Bacterial Propeptide Can Be Divided into Three Evolutionarily Conserved Regions

The assembly of the eukaryotic CP involves five dedicated chaperones and seven unique α and β subunits. The genomes of bacteria and archaea normally encode one α and β subunit each (Maupin-Furlow et al., 2006), eliminating the need for a specific order of subunits within the rings as well as for the rings relative to each other (Murata et al., 2009). Consistent with this lower complexity, the archaeal and bacterial CPs assemble more readily and without the need for specific chaperones *in vitro* or in *E*. *coli* (Becker and Darwin, 2017), whereas human CP has only been heterologously expressed recently with the need of chaperones (Toste Rego and da Fonseca, 2019). Considering the absence of chaperones in bacteria and the reported role for the propeptides of the β subunits in assembly, we hypothesized that some of the functional roles of eukaryotic assembly chaperones could be performed by the propeptides of the eubacterial β subunits. To assess this, we focused on the propeptide *of Rhodococcus erythropolis* (*R*.*e*.) 20S β subunit Prcβ1 (proteasome component β1). *R*.*e*. has two paralogs of α and β each and it has been repeatedly demonstrated that a functional CP forms with only one α and β present (Sharon et al., 2007, Zühl et al., 1997a, Zühl et al., 1997b). Furthermore, it is amendable to *in vitro* reconstitution and there is detailed structural and biochemical information, allowing us to interpret our results in a structural context (Figure S1) (Kwon et al., 2004, Witt et al., 2006, Zühl et al., 1997a).

**Figure 1:**
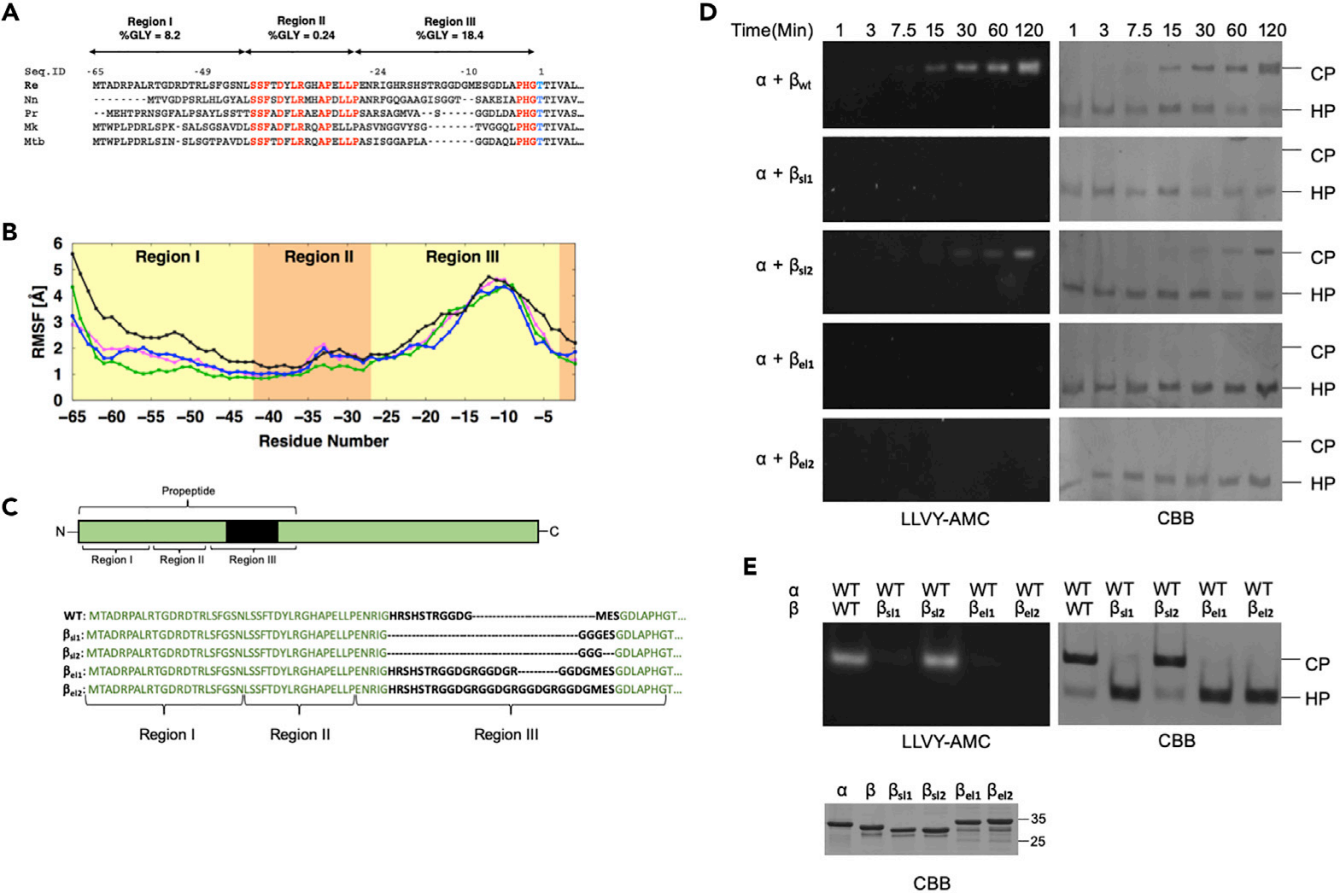
Region III of *Re* β Subunit Is Flexible Loop that Controls HP Dimerization (A) Representative alignment of the N-terminal sequence derived from a multiple sequence alignment of 256 bacterial proteasome β subunits. Sequences shown are derived from *Rhodococcus erythropolis* (*Re*), *Nocardia niwae* (*Nn*), *Prauserella rugosa* (*Pr*), *Mycobacterium kansasii* (*Mk*), and *Mycobacterium tuberculosis* (*Mtb*). Conserved residues are highlighted in red and the active site threonine in blue. The three regions recognized in the propeptide are depicted by arrows with the percentage of glycine residues indicated. (B) Root-mean-square fluctuations (RMSF) calculated from molecular dynamics simulations of the *R*.*e*. propeptide for the three replicates (magenta, blue, and green) and ANTON 2 (black) system are plotted for each amino acid. The background color indicates ordered region (orange) and disordered region (yellow) of the propeptide. (C) Graphical representation of the β subunit of *R*.*e*. with the flexible loop indicated in black. The N-terminal sequence, including the flexible loop (black), is shown for wild-type and mutants β_sl1_, β_sl2,_ β_el1_, and β_el1_. (D) Equimolar amounts of α and indicated flexible loop mutants were reconstituted *in vitro* at 30°C for indicated time points. Samples were separated on native-PAGE visualized by in gel LLVY-AMC assay and CBB staining. (E) Indicated α and β mutants were reconstituted overnight and analyzed as in (D) (top). CBB-stained SDS-PAGE of indicated subunits used for reconstitution (bottom).

To assess potential conservation of properties among bacterial propeptides, we performed a multiple sequence alignment of β subunits from different bacterial species that showed 61% or more sequence identity to the *R*.*e*. β subunit (Prcβ1) sequence (256 unique sequences in total). We chose this cutoff to include *M*. *tuberculosis* and all more closely related sequences. We defined three distinct regions (named I to III) in the propeptide (Figure 1A). Region II is largely identical to the previously described “central box”; it has an average length of 16 amino acids and corresponds to residues from −42 to −27 in the *R*.*e*. Prcβ1 sequence (propeptide residues are labeled with negative numbers, with residue −1 being just N-terminal of the propeptide cleavage site). Region II has a well-defined crystal structure and functions in ring formation by allowing a single β subunit to interact with two α subunits (Kwon et al., 2004). It is highly conserved among bacterial species at the sequence level (Figure 1A). Region I has an average length of 18 amino acids (residues −65 to −43 in *R*.*e*.) and is not strongly conserved across bacterial taxa. Region III has an average length of 17 amino acids (−26 to −1 in *R*.*e*.). Like region I, region III did not show any obvious sequence conservation. However, our analysis indicates region III is highly enriched in glycine residues with 18.4% glycine, compared with 0.24% for region II and 8.2% for region I. The latter is close to the average glycine composition for globular proteins (∼8% [Creighton, 1983]).

The enrichment of glycine in region III is highly unlikely to have arisen purely by chance (p = 3.9×10^−142^, hypergeometric test), indicating that there is likely some evolutionary pressure to maintain it. This suggests that flexibility of this region may be important for its function. Nevertheless, biochemical and structural analyses to date have not identified any clear function for either region I or III.

### Propeptide Region III Regulates HP Dimerization

Glycine residues generally disrupt α helices and β sheets and are more common in loops between secondary structural elements and as flexible linkers between protein domains or regions (Imai and Mitaku, 2005, Levitt, 1978). Therefore, we postulated that there has been an evolutionary pressure to generate a flexible, disordered loop between the active site and the structured region II of the propeptide. To gain insight into this flexibility we used molecular dynamics (MD) simulations of the HP from *R*.*e*. We performed two distinct sets of simulations of the HP structure (generated from PDB:1Q5R). One set used nanoscale molecular dynamics (NAMD) to simulate three separate replicate systems, each running independently for 100 ns per replicate. The other set used the Anton 2 machine (Shaw et al., 2014) to simulate the same HP structure for a total of 2 μs. We characterized and plotted the flexibility of the propeptide in these simulations by calculating root-mean-square fluctuation (RMSF) for the backbone atoms (C, O, N, and Cα) in the HP simulations (Figure 1B). RMSF is a metric used for determining the atomic mobility per residue averaged over time. As expected, region III showed considerable flexibility and a high RMSF, consistent with a highly disordered loop (Figure 1B). Interestingly, in the MD simulations, part of region III resided for an extended time outside of the HP (for example, see Video S1). The presence of propeptide density outside the HP could potentially cause a steric clash with a second HP, which would need to occupy this space during dimerization. Propeptide density observed outside of the barrel in cryo-EM structures of the *M*. *tuberculosis* HP also points to a similar role (Li et al., 2010).

Video S1 Molecular Dynamics Simulations of the *R.e.* Propeptide, Related to Figure 1 Video of the wild-type proteasome structure (1Q5R) showing the movement and flexibility of the propeptide. This is a 2-μs second trajectory run where the alpha subunits are colored in gray, beta subunits are colored in orange, and the propeptide is shown in red (spheres). For this movie every 20th frame has been selected, i.e., every frame corresponds to 4.8 ns run and it has been rendered using the VMD-movie making tool.

To understand the role of region III, we designed mutants that altered the length of the flexible loop (Figure 1C). Two mutants, β_sl1_ and β_sl2_, were created with shorter flexible loops based on the minimal theoretical length needed to span the distance between region II and the remainder of the β subunit in the crystal structure (Kwon et al., 2004). Two other mutants, β_el1_ and β_el2_, were created with extended flexible loops with the idea that these loops would spend more time in the space outside the HP and thus be more effective at sterically blocking HP dimerization. We attempted to maintain (putative) flexibility by including glycine in the modified loops. After heterologous expression in *E*. *coli*, purified mutant forms were reconstituted with α subunits. Native gel analyses showed that all were able to quickly and efficiently form the HP, indicating the flexible loop in region III is dispensable for HP formation and that region II is likely properly folded and positioned to allow the association of β and α subunits (Figure 1D) (Kwon et al., 2004). However, all four loop mutants showed strongly reduced efficiency in dimerization with less (β_sl2_) or no (β_sl1_, β_el1_, and β_el2_) detectable active CP being formed after 120 min. This suggests that there is some evolutionary optimization in the length and/or composition of this region (Figure 1D). Upon overnight incubation, we detected a very small amount of active CPs for the β_sl1_, β_el1_, and β_el2_ mutants (Figure 1E). The observed enzymatic activity correlated with the amount of full CP formed. This suggests that these three mutants are severely compromised in HP dimerization. To test the ability of these mutants to form full CP, we mixed them with a form of the WT β where the active site threonine was substituted to alanine (β_TtoA_) rendering this mutant catalytically dead but with a WT propeptide sequence. Here, any CP activity observed upon mixing the two forms of β must be derived from the flexible loop mutants. This would also indicate that (1) those mutants are successfully incorporated into the CP and (2) they retain the capacity for autocatalytic cleavage of the propeptides. When β_sl1_ was reconstituted with α and β_TtoA,_ we observed a substantial amount of active CP with a 1:1 ratio of β_sl1_:β_TtoA_ (Figure S2A). Similar experiments with the extended loop mutants (β_el1_ and β_el2_) showed very different results (Figure S2B). The 1:1 ratio showed very little CP. Counterintuitively, when reducing the relative amount of β_el1_ or β_el2_ mutant compared with the inactive β_TtoA_ (1:7) we saw increased amounts of CP and activity, indicating these mutants dramatically inhibit dimerization. Thus, although the levels of β subunits with WT active site residues (i.e., β_el1_ or β_el2_) were lower, the CPs showed increased activity, which indicates that β_el1_ or β_el2_ mutants undergo efficient autocatalytic processing when they are successfully incorporated into CPs. In sum, region III affects the HP dimerization, but it has no apparent role in HP formation or in the autocatalytic processing of the β propeptide during CP maturation.

**Figure 2:**
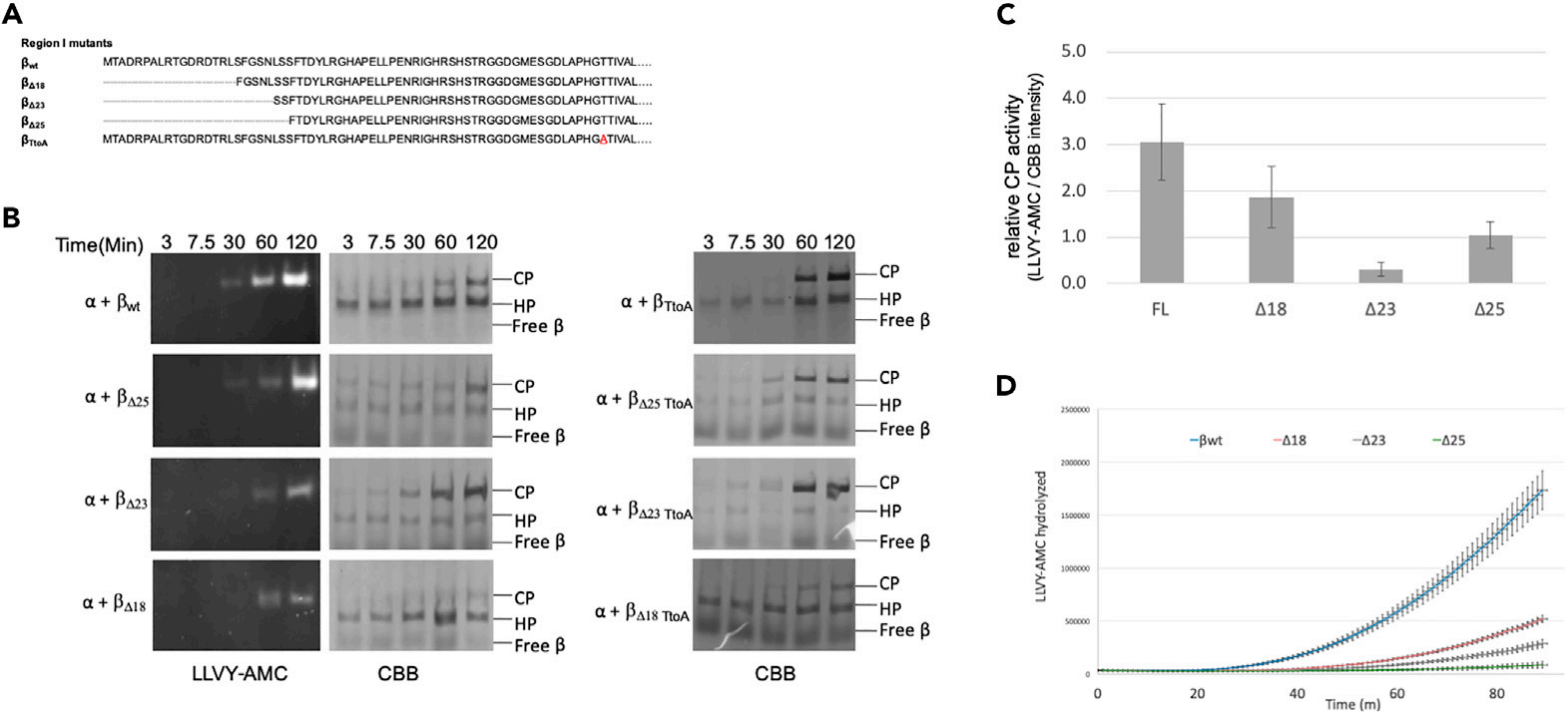
Region I of *R*.*e*. β Propeptide Inhibits HP Dimerization and Promotes Autocatalytic Activation (A) Alignment of N-terminal sequence of the region I in wild-type and mutants of β. (B) Equimolar amounts of wild-type or mutant proteasome subunits were reconstituted at 30°C for indicated time periods and analyzed as in (1D). Data indicate that the truncations exhibited slower maturation as there is less CP activity despite the more rapid formation of CP. LLVY-AMC activity assays for TtoA mutants are not shown as these mutants lack hydrolytic activity. (C) Quantification of LLVY-AMC and CBB for region I mutants carrying active site T in Figure 2B. The ratio of AMC florescence signal (reflects the activity) and the amount of assembled CP (as CBB scanned intensity) for the different forms of β subunits is shown here. Data here are the average of three independent experiments and are shown with standard error of mean (SEM, n = 3). (D) Equimolar amount of α was reconstituted with indicated β subunits in the presence of LLVY-AMC. Accumulation of fluorescent AMC over time was monitored at 30°C. Data are the average of seven independent experiments and are shown with standard error of mean (SEM, n = 7).

In the reconstitution experiments with an equimolar mixture of β_el1_ and β_TtoA_, the HPs that form will have a distribution in the number of β_el1_ subunits versus β_TtoA_, which should be centered on the 1:1 ratio if both are incorporated with the same efficiency into HPs. Based on our model, the extended region III would interfere with dimerization. Hence, the HPs that failed to dimerize should be those that are enriched in βs with an extended loop (β_el1_ or β_el2_). On the other hand, HPs with lower levels of, for example, β_el1_ relative to β_TtoA_ should be preferentially incorporated into CPs. To test this, we performed a reconstitution using β_el1_ in combination with β_Δ23 TtoA_ (which is the N-terminal deletion mutant in the β_TtoA_, see Figure 2A). The truncation in β_TtoA_ allowed us to determine the ratios of the two different βs in CP and HP bands by using native SDS-PAGE 2D gel analyses (Figure S2C). The quantification of the relative abundance of β_Δ23 TtoA_ to β_el1_ or β_el2_ mutant in HP and CP showed that indeed the HPs that failed to form CPs were enriched in the mutant with extended flexible loops (β_el1_ and β_el2_) (Figure S2D).

### Role of Region I in Autocatalytic Processing

*In vitro* reconstitution assays have shown that β subunits with large truncations from the N-terminal region I had defects in HP assembly. These mutants accumulated as either assembly intermediates or free subunits (Sharon et al., 2007). This indicates that the poorly conserved region I might be important in assembly. It has been noted before that poorly conserved regions can play important roles in proteasome assembly with β5 in eukaryotes (Li et al., 2016). To assess the role of region I, we generated three N-terminal truncation mutants in β or β_TtoA_ (Figure 2A). Reconstitution of these mutants showed a faster formation of CP (Figure 2B). This effect was most pronounced in the Δ23 and Δ25 truncations, where CP could be observed with CBB-stained native PAGE gels within 3 min of starting the reconstitution (Figure 2B, middle and right panels). Although there was a more rapid rate of dimerization, we surprisingly saw a delayed or reduced proteolytic activity (Figure 2B, left panels). We also quantified these gels and determined the ratio between 7-Amino-4-Methylcoumarin (AMC) fluorescent signal (which reflects activity) and the amount of assembled CP (as Coomassie Brilliant Blue (CBB) scanned intensity) (Figure 2C). With wild-type CP this number is higher than that of mutants, indicating wild-type CP has already gained full activity. For the N-terminal truncations this number is much lower, indicating the same amount of CP produced less AMC. This is consistent with the idea of slower maturation in the region I mutants. Consistently, in solution, real-time LLVY-AMC hydrolysis was dramatically reduced in all three region I mutants compared with β_wt_ (Figure 2D). This is particularly interesting as generally the dimerization of HPs appears to be tightly coupled with the activation of the CP. For example, despite extensive efforts, pre-holocomplexes (i.e., CP containing all unprocessed propeptides) have not been observed (Witt et al., 2006), unless mutants were used to block propeptide processing completely, such as K33A (Kwon et al., 2004) or T1A (Figure S1C).

**Figure 3:**
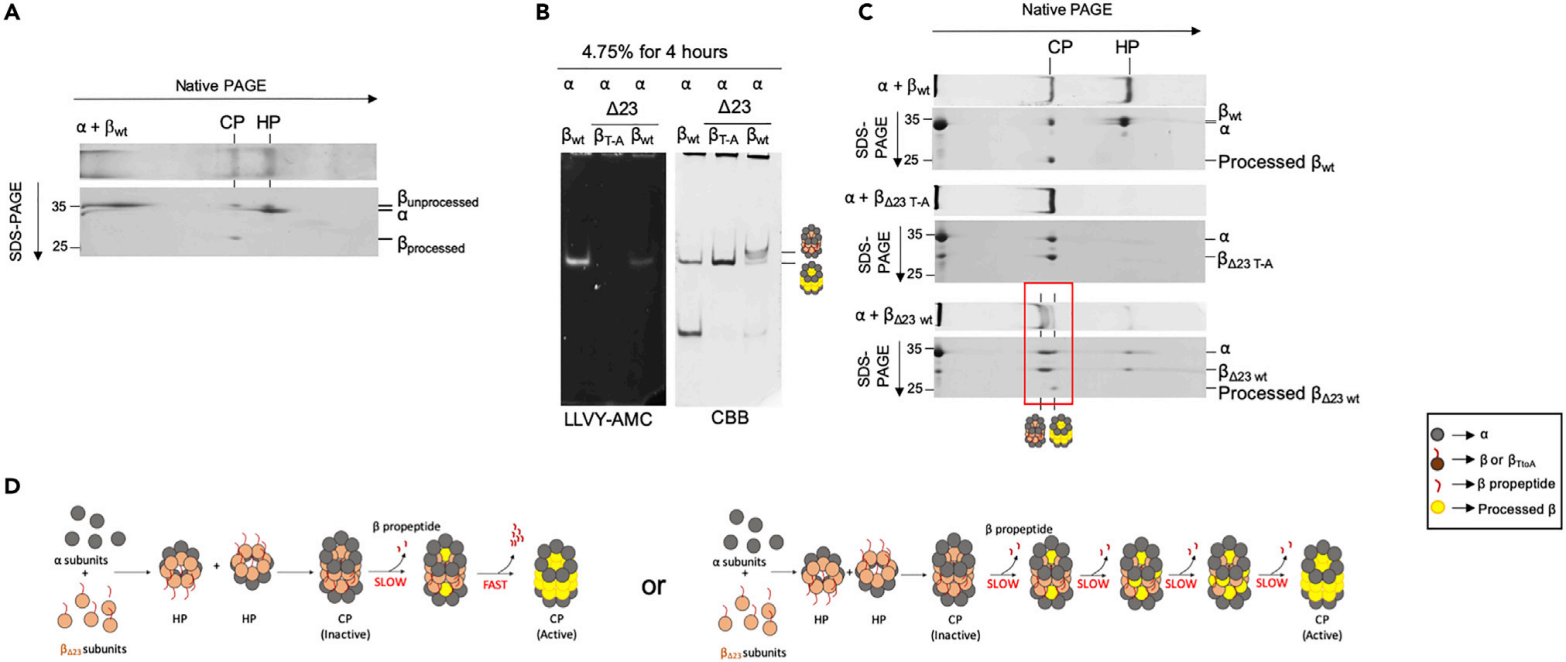
Cooperativity in the Activation of CP. (A) Reconstitution of α with β_wt_ was evaluated by 2D native SDS PAGE. Top panel shows CBB stain of one lane from a native PAGE gel. Lower panel shows 2D-PAGE analyses where the native PAGE lane was separated on a second dimension of SDS-PAGE. The second dimension can reveal the MW of the polypeptides found in native PAGE CP and HP bands. (B and C) (B) Reconstitution with indicated subunits (2 h, 30°C) were separated on native-PAGE (4.75% gel for 4 h) and visualized for hydrolytic activity using LLVY-AMC (left) and protein using CBB (right). (C) Lanes from (B) were also excised prior to visualization and separated on a second dimension of SDS-PAGE and described in (A). (D) Slow and independent autocatalysis of CP, as indicated in the model of the right, should result in the presence of many intermediate forms of partial CP maturation. Since this is not observed, we propose a model that incorporates cooperativity in the autocatalytic processing of the propeptides resulting in an accumulation of either immature or mature CP (Left).

The slow activation of the CP for region I mutants suggests that the N-terminal region plays a role in efficient autocatalytic cleavage of the propeptide from β. To test this, we analyzed reconstitutions on 2D gels where native PAGE was followed by SDS-PAGE, allowing us to determine the molecular weight (MW) of HP and CP components (Figure 3A). For wild-type α and β, two bands of different MW were visible in the area where HP migrates, namely, full-length tagged α (31 kDa) and full-length tagged β (32 kDa). For the CP, we again saw full-length α but observed a shift in MW of β to ∼25 kDa, consistent with the molecular weight of β after the propeptide has been removed. When we used the β_TtoA_ mutant in our reconstitutions, both HP and CP were composed of full-length α and β (Figure S3). Reconstitution of α with β_Δ23_ resolved under optimal conditions on native-PAGE showed three distinguishable migrating CP bands (Figure 3B). The top two bands migrated very closely and were not distinguishable on 2D gels, suggesting they are identical in composition and are either a staining artifact or two different conformations of the same complex. Only the faster migrating species had proteolytic activity, indicating this was matured CP, whereas the upper bands were pre-holoenzyme. Pre-holoenzyme has never been observed with autocatalytically competent mutants. The 2D gel electrophoresis of these samples (Figure 3C, lower right panel) showed two distinct spots for β associated with these two CP forms, which are distinct in both dimensions. The slower migrating CP species contained a spot of ∼28 kDa corresponding to unprocessed β (confirmed by mass spectrometry to be 28,545 Da). The faster, active CP species shows a spot of ∼24 kDa corresponding to processed β (24,030 Da by mass spectrometry). α Subunits are visible as a single broad spot that encompasses both forms of CP. Thus, truncation of the region I results in two distinct CP populations, one without proteolytic activity and exclusively composed of β with propeptides and one with proteolytically active CP where all β’s had undergone autocatalytic processing.

### Cooperativity in Maturation

The existence of these two forms of CP is unexpected when considering reports that the maturation of CP in bacteria, archaea, and eukaryotes depends on the autocatalytic processing of the β propeptide for each active site (Baumeister et al., 1998). If all sites undergo proteolytic processing independently, then under conditions of slow maturation as we identified here, one would expect to see an array of intermediate species where some β’s are processed while others are not (Figure 3C, model on the right). However, we see a sharp delineation with CP populations either all containing propeptide or none at all (Figure 3B). This suggests the existence of a cooperative phenomenon during the maturation process. One possibility is that all active sites undergo autocatalytic processing simultaneously, e.g., as a result of simultaneous global conformational changes. Another explanation is that activation is a two-step process involving a slow initial step where one β subunit undergoes autocatalytic processing. This slow initial step would then be followed by a very rapid activation of all other active sites through an unknown mechanism that depends on the presence of one active β subunit (Figure 3D, model on the left).

Interestingly, it has been shown in eukaryotes that β subunits can cut the propeptides of neighboring β subunits. Indeed, since only three of the five β subunits with cleaved propeptides actually have functional active sites, eukaryotic β’s must and are able to cleave propeptides from other subunits, this cross-cutting can happen within a ring as well as between the two β rings (Chen and Hochstrasser, 1996, Groll et al., 1999, Schmidtke et al., 1996, Seemuller et al., 1996). However, the functional significance of this cutting is not well understood. We hypothesized that, if such cross-subunit cutting is conserved in bacterial CPs, it provides a potential mechanism for the cooperativity that we observed. Specifically, we could envision that an initial autocatalytic activation of one β subunit allows this active site to cut the neighboring propeptides. These cross-cuts cannot be at the site where autocatalytic cleavage occurs (owing to the distance between active sites, which is more than 24 Å) but could happen within the propeptide sequence. The flexible region III seems like the most likely place for this to occur. A cleavage here would disconnect the structured part of the propeptide, which is physically constrained by binding to α subunits, from the part that needs to be properly positioned for autocatalytic cleavage. Cross-cutting would thus eliminate physical constraints that might slow activation. In this scenario, a single initial activation event would lead to rapid subsequent cleavage of all the propeptides in the CP.

### Cleavage of Propeptides by Neighboring Beta Subunits

**Figure 4:**
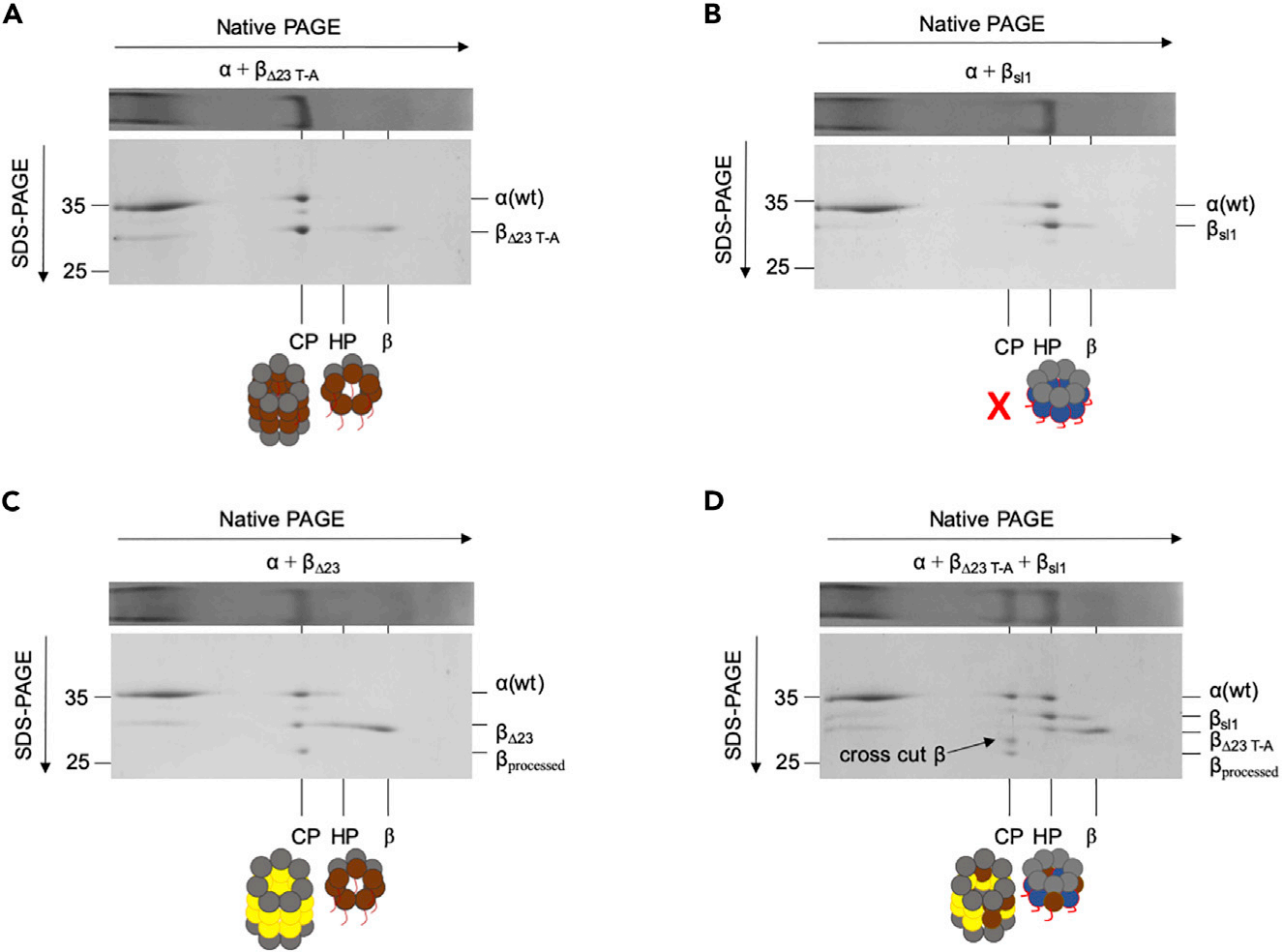
Active β Subunits Can Cleave the Propeptide of a Neighboring β Subunit Four panels show 2D-PAGE analysis as described in (3A) for α reconstituted with β_Δ23 TtoA_ (A), β_sl1_ (B), β_Δ23_ (C), or an equimolar mixture of β_Δ23 TtoA_ and β_sl1_ (D). All subunits were reconstituted at 4 μM concentrations, except in (D) where each β subunit was present at 2 μM for a total of 4 μM β. (A) and (B) serve as controls to show cleavage (indicated by arrow) observed in (D) requires the combination of βs. (C) was used to determine the size of completely processed β subunit present in an active CP.

To test if the cross-cutting by β subunits is conserved, we combined equal amounts of β_Δ23 TtoA_ and β_sl1_ and reconstituted with α (Figure 4). We used β_Δ23 TtoA_ as it cannot undergo autocatalytic cleavage and the truncation enables us to distinguish it on 2D SDS PAGE by size (Figures 4A and 4C). β_sl1_ fails to form CP when reconstituted by itself with α for 2 h (Figure 4B). Thus, combining these two forms of β ensured that all active CP was derived from a heterogeneous mixture of both β forms (Figure 4D). Our analyses showed that the active CP was composed of α, completely processed β, and a novel band, which we refer to as “cross-cut β” (Figure 4D arrow). The partially processed β subunits remained present even after extended incubation period (data not shown). The cross-cut β must represent β_Δ23 TtoA_ subunits that have been processed by a neighboring β_sl1_ present in the CP. The neighboring site would be unable to remove the complete propeptide; hence, we see a higher MW band as compared with fully processed β subunits. The occurrence of this processing of β_Δ23 TtoA_ by β_sl1_ shows that cross-processing by neighboring β subunits is evolutionarily conserved between bacteria and eukaryotes and provides a potential mechanism for the cooperativity we observed in the activation process.

**Figure 5:**
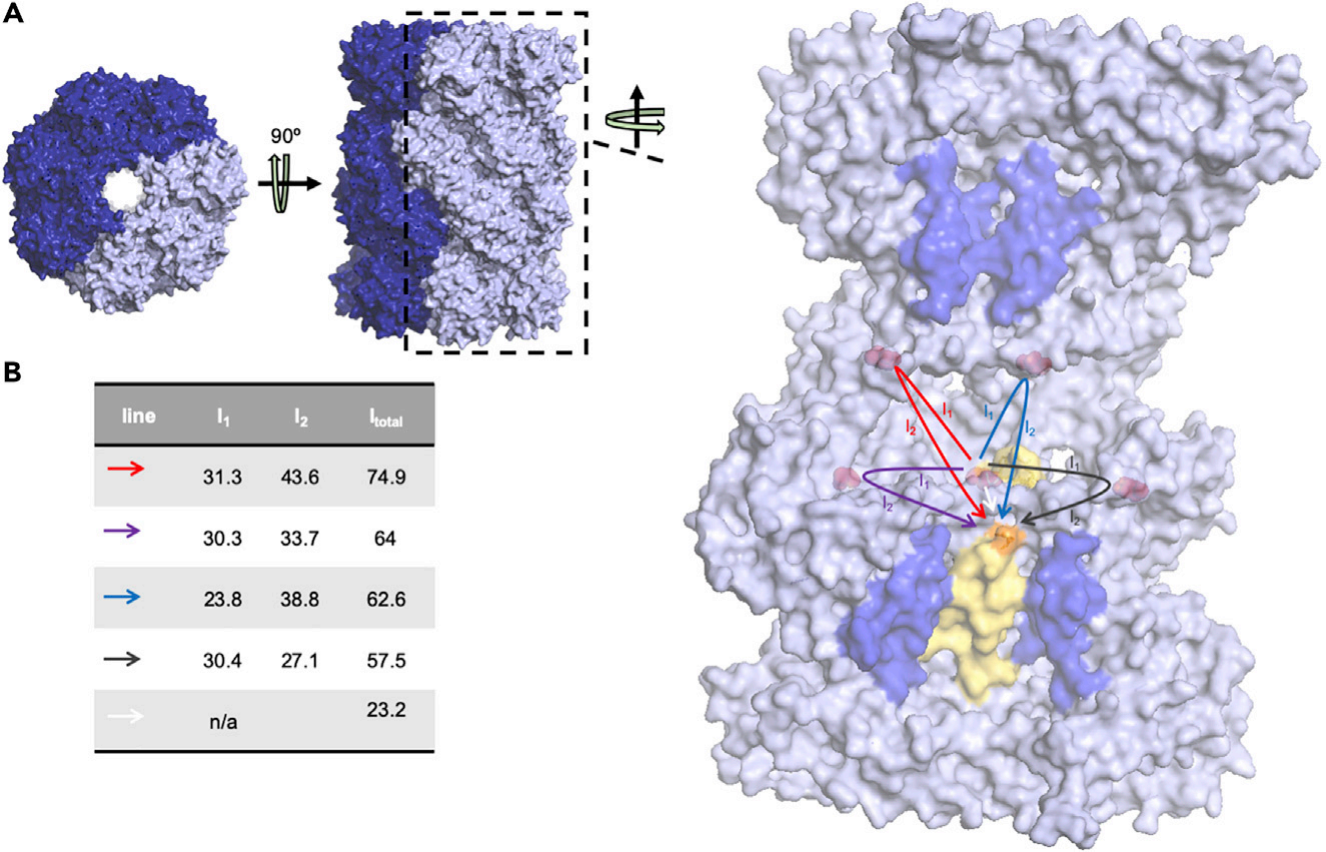
Distance between Active Sites Relative to Cleavage Sites Space filled rendering of the structure of *R*.*e*. CP (PDB:1Q5R), visualized from two angles and an enlarged view of a subset of residues. The later provides an interior view exposing CP active site threonines (red) and the structured elements of several neighboring propeptides (dark blue and one in yellow). The orange residue indicates arginine −24, which is the last structured amino acid of region II (yellow). The flexible region III spans from the orange R to the active site in the center. The white arrow depicts the straight distance, but the observed cleavage by neighboring active sites requires region III to engage with neighboring active sites and thus span the distances (l_1_) between the central active site and the different neighboring active site. I_2_ indicates the distance from the neighboring active site T to R^−24^. Total required distance l_total_ is indicated with colored arrows. (B) Table of distances between active site and structured propeptide when propeptide would engage with specific neighboring sites as described in (A).

To accurately determine the size of “cross-cut β” and the exact site in propeptide region where the cleavage occurred during the cross-cutting described above, we determined the mass of proteins present in the reconstituted samples using MALDI-TOF (Figure S4A). As expected, we obtained peaks corresponding to the molecular weight of the subunits used for reconstitution and a strong peak corresponding to the molecular weight of β with completely processed propeptide (25,195.88 Da). Instead of obtaining a single peak for the partially processed band, we saw four peaks each separated in molecular weight by the equivalent of approximately two amino acids. Based on the molecular weight of these peaks, we determined the likely sites of cross-cutting (Figures S4B and S4c). We obtained a similar result when we repeated the reconstitution and analyses using β_sl1_ and β_TtoA_ (data not shown). Interestingly, our analysis indicates that the cleavage we observed occurs in the flexible linker region III (Figure 5A). The identified cleavage sites are consistent with distances between active sites within the crystal structures (Figures 5B and 5C). In all, these data show that, in the CP, the propeptide can be cross-cut in the flexible region III by a neighboring β subunit.

### Propeptide Cleavage across HPs

**Figure 6:**
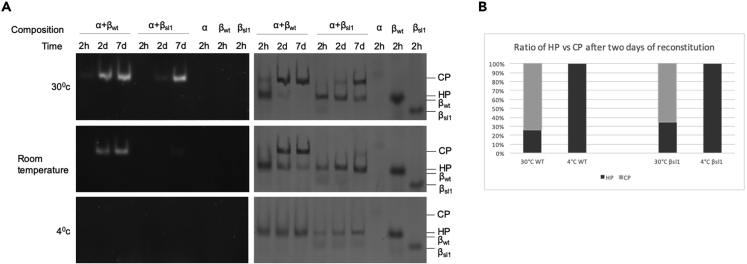
Assembly of Proteasomes Can Be Controlled by Reconstitution at Different Temperatures (A) Reconstitution of indicated subunits was incubated at different temperatures (30°C, room temperature [~21°C], or 4°C) for 2 h, 2 days, or 7 days. Samples were separated by native PAGE and visualized for peptidase activity (using LLVY-AMC), followed by protein staining using CBB. (B) Quantification of the ratio of HP versus CPs at 30°C and 4°C. The concentrations of HP (black) and CP (gray) were determined from quantitative analysis of native gel bands using GeneTools (Version 4.03.05.0) from SynGene.

**Figure 7:**
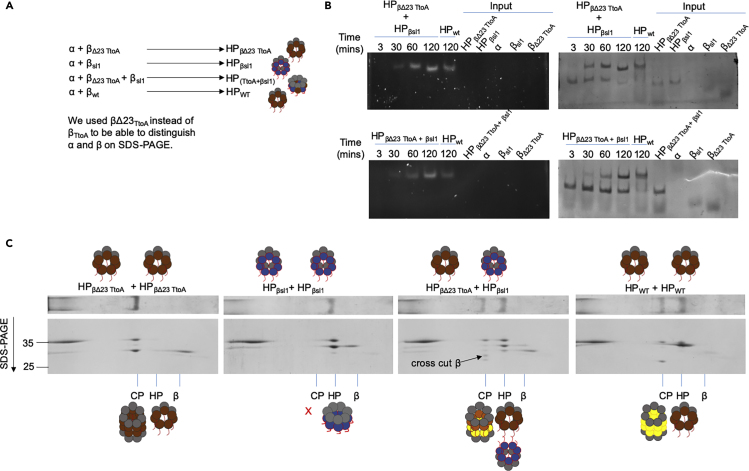
Active β Subunits Can Cleave the Propeptide of a β Subunit across the Dimerizing Interface (A) Schematic representation of the HPs formed by reconstituting α with indicated β mutants at 4°C. Reconstitutions were incubated overnight at 4°C to maximize HP and minimize non-incorporated subunits. (B) The dimerization of HPs was studied by mixing preformed HPs of indicated type and incubating samples over a 2-h time course at 30°C. CP formation and activation were monitored after native PAGE by peptidase activity assays using LLVY-AMC and subsequently CBB staining of gels. (C) 2D-PAGE analysis of a 2-h reconstitution mixture consisting of indicated HPs was done by first separating on native gel followed by SDS-PAGE and are stained with CBB. Arrow indicates cross-cut of β_Δ23 TtoA_ across the dimerizing interface by active β present in the other HP.

The distances between active site threonines within a β ring are comparable with the distances between active site threonines present across two different HPs. This would suggest that cross-cutting of a propeptide can occur between two adjoining HPs, across the dimer interface. To test this, we developed an assay where we could form stable HPs and control their dimerization process, by conducting the reconstitution at different temperatures (30°C, room temperature [∼21°C], and 4°C) over different times (Figure 6A). At 30°C, we observed wild-type HPs and active CPs as early as 2 h followed by a progressive increase in active CP and nearly complete absence of HPs after 2 days of reconstitution (Figure 6B). β_sl1_ has a comparatively slower dimerization rate with active CPs forming after 2 days. The reconstitution at room temperature showed similar but slower dimerization rates. Interestingly, the reconstitution done at 4°C showed no dimerization even after an incubation of 7 days. We took advantage of this inability to dimerize at 4°C to generate HPs containing specific β mutants, HP_βsl1_ (HP containing α and β_sl1_; does not dimerize with itself) and HP_βΔ23 TtoA_ (HP containing α and β_Δ23 TtoA_; does not form active CPs). Using these in reconstitution mixtures at 30°C allowed the analyses of inter-HP interactions during the assembly process (Figure 7A). Reconstituted HP_βsl1_ with HP_βΔ23 TtoA_ at 30°C was analyzed by native-PAGE over a 2-h period (Figure 7B). We saw an increased peptidase activity over time, indicating the dimerization of two different HPs. 2D analysis of reconstitution showed the presence of the “cross-cut β” form as seen previously (Figure 7C). MALDI-TOF analyses confirmed the partially processed β’s of similar MWs as seen previously (data not shown). Thus, our data show that β subunits can be processed across the HP dimer interface as well within a ring.

Utilizing this new two-step reconstitution assay, we also re-evaluated dimerization rates of HPs formed by other region I mutants and obtained same results as seen in Figure 2B (Figure S5A). Similarly, consistent with what was seen in Figure 1D, when we used β_el1_ or β_el2_ to first make HPs and tested how they dimerize with themselves, we observed a failure to dimerize (Figure S5B). However, on reconstitution of HP_βel1_ with HPs that were formed with β_Δ23__TtoA_ (HP_βΔ23_ _TtoA_), we observed the formation of active CP. Since HP_βel1_ or HP_βel2_ does not dimerize by itself and CP from β_Δ23__TtoA_ is inactive, the CPs that showed activity must be derived from HP containing β_el1_ or β_el2_ that dimerized with HP containing β_Δ23__TtoA_. The formation of active CP here is surprising because HPs that are formed by mixing of β_el1_ (or β_el2_) and β_Δ23 TtoA_ in a 1:1 ratio failed to dimerize (Figure S5B, lane 1 and 4). This difference suggests that the single extended loop by itself can be accommodated in dimerization but dimerization is inhibited when extended loops are present on opposing HPs that meet. This is consistent with our model that the extended flexible region III can regulate dimerization by populating states outside of the HP where it can sterically clash with a dimerization partner.

## Discussion

The assembly of the CP starts with the formation of HPs. This is followed by two key events, the dimerization of HPs and the processing of propeptides to form active CP. In eukaryotes the dimerization is controlled both positively and negatively by some of the propeptides as well as specific assembly chaperones (Barrault et al., 2012, Ehlinger and Walters, 2013, Kock et al., 2015, Park et al., 2013, Roelofs et al., 2009, Satoh et al., 2014, Singh et al., 2014, Wani et al., 2015). As far as we know, bacterial genomes do not encode assembly chaperones, but we report here that a flexible loop in the propeptide of *R*.*e*. β subunits also has a major impact on dimerization. We identified mutants that show rapid dimerization and delayed autocatalytic processing, showing for the first time that these two steps are distinct and allowing us to show the existence of the pre-holoenzyme as an intermediate in a complex capable of autocatalysis. Thus, dimerization by itself is not the trigger for autocatalysis. Our data indicate there is a cooperative process that coordinates autocatalysis, potentially involving propeptide trimming of neighboring β subunits by an initially autocatalytically activated β subunit.

### Effect on HP Dimerization

Our data showed that region III (see Figure 1) of the *R*.*e*. β propeptide can negatively regulate the dimerization of HPs. This is a property strikingly similar to the role of several proteasome chaperones, as they sterically block steps in assembly (Barrault et al., 2012, Ehlinger and Walters, 2013, Kock et al., 2015, Park et al., 2013, Roelofs et al., 2009, Satoh et al., 2014, Singh et al., 2014, Wani et al., 2015). Particularly interesting is Ump1, a eukaryotic chaperone that assists CP assembly by inhibiting the premature dimerization of HPs (Li et al., 2007, Ramos et al., 1998). Our MD simulation data suggest that the residues in region III provide for a highly flexible loop that extends out of the HPs on the dimerization interface. Manipulating the length of this loop dramatically reduced HP dimerization rates when tested *in vitro*, whereas there was no apparent effect on HP formation. The inability to form CP, even after overnight incubation, for most mutants suggests a conservation of optimal size or charge on the flexible disordered loop to allow for proper repositioning of the propeptides during assembly. Although both the longer (el1 and el2) and shorter (sl1 and sl2) β mutants were compromised in dimerization as compared with wild-type, the reconstitution experiments done using a mixture of these mutants with β_TtoA_ showed that the mutants with shorter flexible loops were fundamentally different from those with extended flexible loops. The smaller flexible loop mutant β_sl1_, for instance, exhibited a severely reduced ability to dimerize by itself but assembled efficiently when reconstituted with a normal-length region III as in β_TtoA_. This might indicate a role for specific flexible loop interactions in bringing the dimerization interfaces together. Interestingly, we found that there is little to no inhibitory effect on the activation step in this mutant. On the other hand, the extended flexible loops of β_el1_ and β_el2_ showed little dimerization when mixed in a 1:1 ratio with β_TtoA_, and decreasing the extended loop mutants in the ratio resulted in more CP activity. Consistently, we observed that the stalled HP was enriched in mutants with the extended flexible loop, suggesting that these mutants inhibit dimerization when they extend out from the HP at the dimerization surface. Interestingly, some recent data suggest the eukaryotic propeptide can also extend out from the HP structure, since the yeast β5 propeptide can be cross-linked to β4 lysine 28, which faces outside of the HP (Kock et al., 2015). These results thus strengthen our model of conservation of the length of flexible loop in β subunits and the function of beta propeptides in regulating dimerization rates.

### Competent Pre-holocomplexes

Studies in *R*.*e*. mutants have suggested a mechanism where the assembled proteasomes mature into active proteasomes following a switch (Witt et al., 2006). Here, the interactions between two helices of the dimerizing HPs (H3 and H4) drive the positioning of specific loops (S2-S3) of β subunits to act as an activation switch by allowing the cleavage of propeptides from β subunits. When we introduced N-terminal truncations in the β subunit propeptide Region I, we unexpectedly observed a slower maturation of the CP that cannot be readily explained based on our current understanding of CP assembly. These findings highlight a positive role for region I in stimulating the autocatalytic processing of the β propeptide. These mutants also allowed us to capture, for the first time, the pre-holoproteasome complex containing β subunits that are fully competent for autocatalysis. This complex showed a slower migration on native gels compared with the CP formed by catalytically dead β subunits (T1A or K33A [Kwon et al., 2004, Witt et al., 2006]), suggesting that it is a distinct structure different from previously observed and crystalized structures. Early studies postulated the autocatalytic cleavage of β to be a rate-limiting step (Zühl et al., 1997a, Zühl et al., 1997b), which would imply that the pre-holoproteasome is a relatively abundant intermediate. However, this intermediate has always remained elusive when using autocatalytically competent forms of β (Sharon et al., 2007, Witt et al., 2006), and propeptide-containing CP crystal structures all from autocatalytically defective mutants are very similar to structures of the active CP.

Based on the crystal structure, region I is expected to extend in the direction away from the loop region mentioned above and toward the gate region of the α ring. A similar direction has been observed for the β5 propeptide in yeast, which can be cross-linked to α6, and thus this propeptide seems to be oriented toward the α ring as well (Kock et al., 2015). Nevertheless, deletion of the N-terminal portion of the β5 propeptide also caused maturation defects (Li et al., 2016). Considering the orientation of this propeptide region, any mechanism that allows the N-terminal portion of the propeptide to stimulate maturation would involve long-distance allosteric changes. Such allosteric pathways have been proposed for cylindrical proteases like CPs (Huber et al., 2016, Kleijnen et al., 2007, Shi and Kay, 2014, Wani et al., 2015). Alternatively, we could envision that the extra mass from the propeptide in the tight cavity of the CP might destabilize the propeptide structures that have formed in HP, thereby facilitating the proper orientation of the propeptide for autocatalytic processing. In support of this idea, the proper positioning of propeptide residues in the β active site pocket is crucial for propeptide processing (Ditzel et al., 1998, Li et al., 2016).

### Cooperativity

Some of our mutants showed a slow autocatalytic activation. If each of the 14 active sites in one CP underwent activation independently of others, intermediate forms of the CP (e.g., with 50% processed and 50% unprocessed active sites) would be expected (see model in Figure 3D). Our inability to detect these and our observation that these CPs are either completely active or completely inactive suggest that a specific mechanism is responsible for the (almost) simultaneous processing of the propeptides by all β subunits. A possible underlying mechanism that could explain these observations is a two-step process. Here, an initial slow step involves the autocatalytic cleavage of a propeptide from one of the fourteen β subunits in CP. This triggers a second step, where the newly created active site initiates hydrolysis of the propeptides of neighboring β subunits. This hydrolysis cannot be at the active site threonine but instead would be upstream in the propeptide. This is consistent with our observation that one β can cut the flexible loop of a neighboring β propeptide either within the ring or across the HP dimer interface. The cross-processing of propeptides could stimulate autocatalytic cleavage by the β subunit with the truncated propeptide if the truncation removed physical constraints that limited the proper positioning required for autocatalysis of the propeptides (Ditzel et al., 1998, Huber et al., 2016), thus triggering a rapid phase of complete cleavage and activation.

### Evolutionary Perspective

The short-length propeptides found in archaea are not required during assembly. Nevertheless, the longer propeptides found in bacterial species like *R*.*e*. used in this paper have been proven indispensable for assembly. Similarly, in eukaryotes, five of the β subunits retain their propeptides. One proposed function of the propeptides is to protect the active site threonine from N-terminal acetylation (Chen and Hochstrasser, 1996); however, this does not explain two of the eukaryotic propeptides, as they are found on β subunits that have lost a functional active site. Clearly, the presence of these propeptides in eukaryotes has been conserved in evolution. Here we report a functional conservation between bacterial and eukaryotic CP assembly: in both, the dimerization of HPs is tightly controlled. Our observation that the propeptides influence the transition from pre-holoproteasomes to active holoproteasomes is intriguing. This transition is likely to involve a conformational change of the CP, as both molecular species can be separated on native gel. In yeast, there is also a conformational change that triggers maturation and the exchange of immature CP binding from the chaperone Pba1/2 to the Regulatory Particle (Kock et al., 2015, Wani et al., 2015). How this switch and maturation are connected remains poorly understood and has been proposed to involve the degradation of Ump1. Our data suggest the propeptides themselves can be another important factor. It may thus be that the propeptides on the catalytically inactive β6 and β7 subunits in eukaryotes are retained in part because they play a role in this conformational switch.

As mentioned in the Introduction, *Mycobacterium tuberculosis* (*M*.*tb*.), the causative agent of tuberculosis (Tb), is a close relative of *R*.*e*. Tb remains a global health threat, and in particular the emergence of antibiotic-resistant strains of *M*.*tb*. necessitates the development of new drugs to treat this disease. The *M*.*tb*. CP is essential for pathogenicity and is a validated drug target (Lin et al., 2009, Totaro et al., 2017). Thus, the development of drugs that interfere with *M*.*tb*. CP function or formation have the potential to be potent drugs against this disease. A key requirement for a successful drug that targets the *M*.*tb*. CP is the ability to target the bacterial CP but not host proteasomes. The evolutionary perspective presented above suggests that there are unique aspects of bacterial CP assembly that could be leveraged in the development of therapeutics for Tb. In any case, a full understanding of the evolutionary conservation of CP assembly mechanisms between humans and bacteria will be critical to further exploration of CP biogenesis as a drug target.

### Limitations of the Study

- A limitation of the current work is that our assays assume that there is no further processing of the propeptides during electrophoresis at 4°, or, if there is, the processing mechanisms in the gel are not substantively different from those that occur in solution. We believe this assumption is reasonable as we observe species where no CP propeptides have been processed at all, whereas the same assay at 30° would lead to full propeptide processing.
- We used specific mutants that allowed us to expose the existence of cooperativity. However, owing to technical limitations in the time resolution we have not been able to show this occurrence in a wild-type background.
- The occurrence of cross-processing of propeptides provides an intriguing mechanism for the observed cooperativity and could also provide a rationale for the need and existence of propeptides in non-catalytic β subunits in eukaryotes. However, a complete causal relationship remains to be established.

## Methods

All methods can be found in the accompanying Transparent Methods supplemental file.

### Resource Availability

#### Lead Contact

Further information and requests for resources and reagents should be directed to and will be fulfilled by the Lead Contact, Jeroen Roelofs (jroelofs@kumc.edu).

#### Materials Availability

All unique/stable reagents generated in this study will be made available on request, but we may require a payment and/or a completed Materials Transfer Agreement if there is potential for commercial application.

## Data and Code Availability

This study did not generate datasets or code.

## Supplemental Information

Document S1. Transparent Methods, Figures S1–S5, and Table S1
