# Supplementary material for: Cooperativity in Proteasome Core Particle Maturation
Source: iScience. 2020 Apr 22;23(5):101090. doi: 10.1016/j.isci.2020.101090 (PMC7210456; doi:10.1016/j.isci.2020.101090)
Supplement: Document S1. Transparent Methods, Figures S1–S5, and Table S1 [file mmc1.pdf]

iScience, Volume *23*

Supplemental Information

Cooperativity in Proteasome Core

Particle Maturation

Anjana Suppahia, Pushpa Itagi, Alicia Burris, Faith Mi Ge Kim, Alexander Vontz, Anupama Kante, Seonghoon Kim, Wonpil Im, Eric J. Deeds, and Jeroen Roelofs

Supplementary figures:

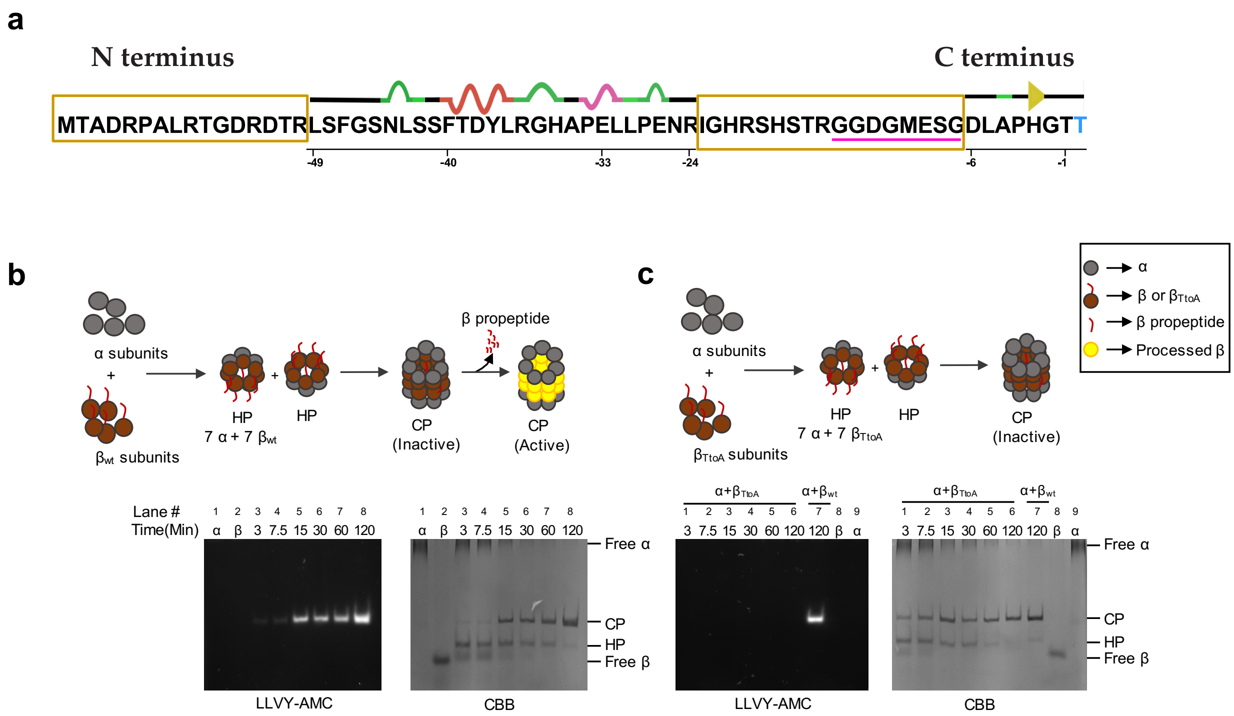

Figure S1. ***In-vitro*** **assembly of the bacterial 20S core particle, Related to Figure 1.**

- **a.** Sequence view of the propeptide sequence of β subunit of *Rhodococcus erythropolis* (*R.e.*) with indicated structure elements adapted from PDB (Berman et al., 2000). The residues with missing electron density are highlighted in yellow boxes and are highly mobile. The flexible region of the propeptide (residues -7 to -14) is underlined in magenta and is a part of the region III that is seen at the half proteasomes interface in the simulations. The active site T (threonine) is shown in blue. This sequence view was created using the PDB (ID:1Q5R) sequence information from the crystal structure of Rhodococcus erythropolis. The secondary structure assignment is based on DSSP algorithm (Kabsch and Sander, 1983); the red structure is an alpha helix, pink structure represents a 3_{10} helix, green structures represent bends and turns and black lines indicate unassigned secondary structures.
- **b.** Graphical representation of the *in-vitro* reconstitution experiment using purified α and $\beta_{wt.}$ Equimolar amount of His-tagged α and β_{wt} proteasome subunits were incubated at 30 °C for indicated time-points and samples were separated on Native PAGE (bottom). The gels were stained for peptidase activity using the fluorogenic peptide suc-LLVY-amc and by Coomassie Brilliant Blue (CBB) to visualize all the protein complexes on the gel. The free α subunits in lane 1 tends to form

aggregates and run on top of gel, whereas β subunits by themselves migrated furthest in the gel and are visible as distinct band (lane 2). The slower migrating band in lane 3 represents the HP that formed rapidly after reconstitution as it lacks LLVY-AMC hydrolytic activity. As the reconstitution progresses, a slower migrating band representing CP appears above HP. The appearance of this band coincides with peptidase activity indicating this is active mature CP. With time, HP disappears and we see more of active CP.

c. Same as b, except an inactive version of β_{TtoA} was used for reconstitution. Mutation of active site T to A renders the mutant unable to remove the propeptide and form active CP complex, also indicated by the absence of activity upon LLVY-AMC assay. Reconstitution mixture containing α and β_{wt} was used as a positive control for peptidase activity upon LLVY-amc assay.

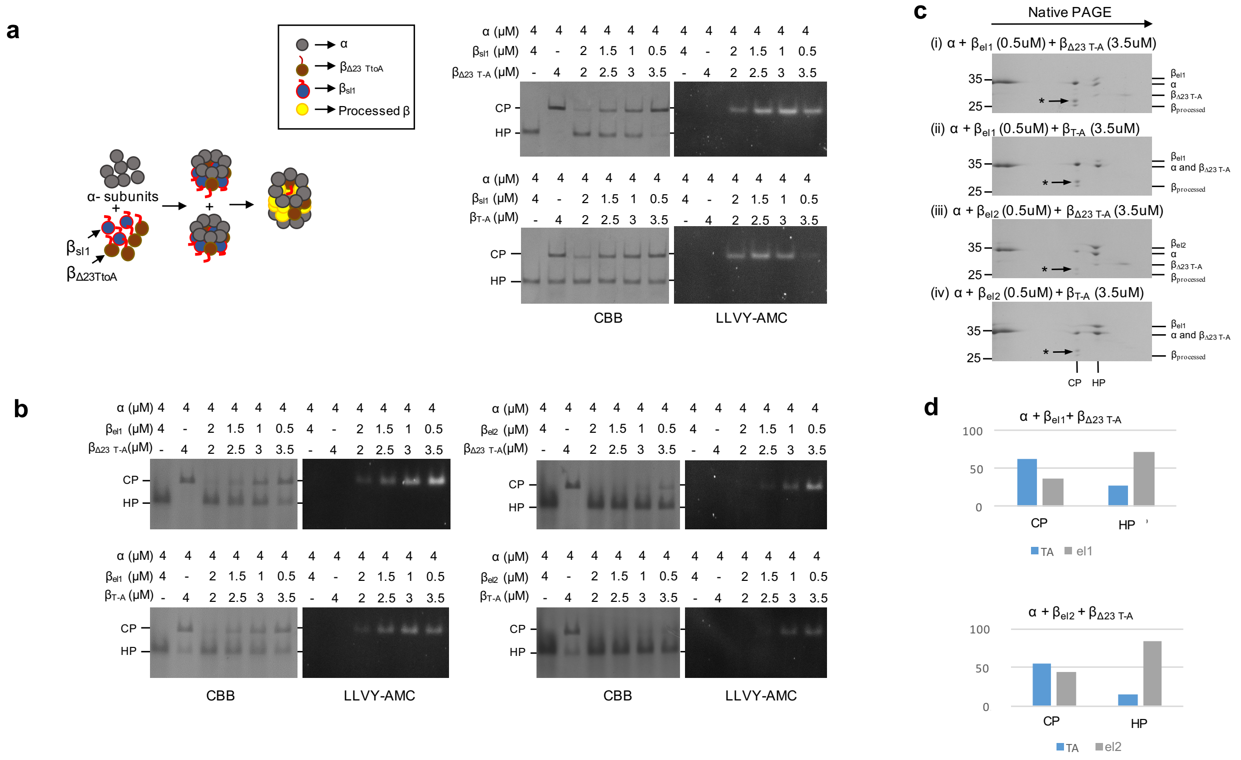

Figure S2. Incorporation of Region III mutants into mature CP upon mixing with β containing complete propeptide, Related to Figure 1.

a. Graphical representation of the assay (left), LLVY-AMC assay and CBB stain of native-PAGE containing samples obtained after reconstituting α with β_{sl1} and β_{TtoA} at indicated molar concentrations for two hours (right).

b. Reconstitution of β_{el1} and β_{el2} with $\beta_{\Delta 23\ TtoA}$ at the indicated molar concentrations, analyzed by LLVY-AMC assay and CBB stain.

c. 2D-PAGE analysis of indicated labelled samples reconstituted at 30°C for 2 hours. The samples were separated on native-PAGE, excised and separated on a second dimension using SDS-PAGE. Arrow indicates the $\beta_{\Delta 23\ TtoA}$ that has been trimmed by other active site. The size is larger in MW then that of proteolytic active β forms, because it lacks the autocatalytic capacity to cleave at the propeptide-protein interface.

d. Quantification of the relative abundance of indicated β subunits in HP and CP seen in set (i) and (iii) of fig S2c. Data indicate that the HPs that do not dimerize are enriched in β with extended loop, while HP with lower levels of β with extended loop preferably dimerize into CP.

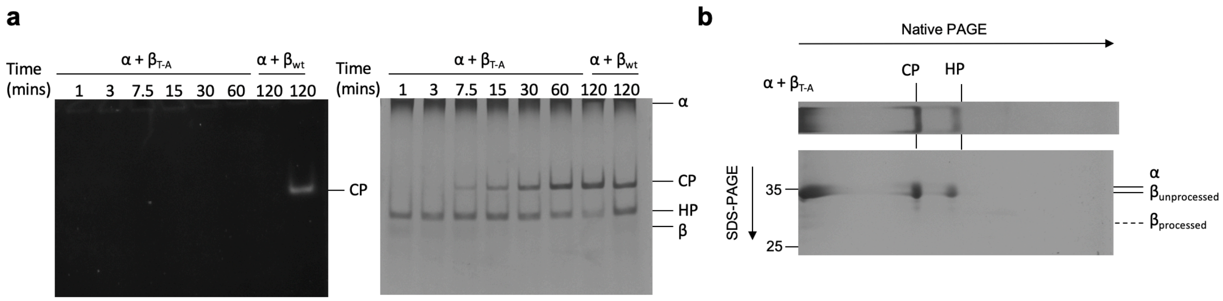

Figure S3. 2-D PAGE analysis of mixture of α and β_{TtoA}, Related to Figure 3.

- **a.** Time-course analysis to monitor the assembly of α and β_{TtoA} reconstitution mixture on 1D native gel.
- **b.** Sample from 1-hour reconstitution was also separated on a second dimension by using SDS-PAGE, as described before. Since β_{TtoA} is an inactive form of β, we do not see any band representing $\beta_{processed}$ in CP.

a

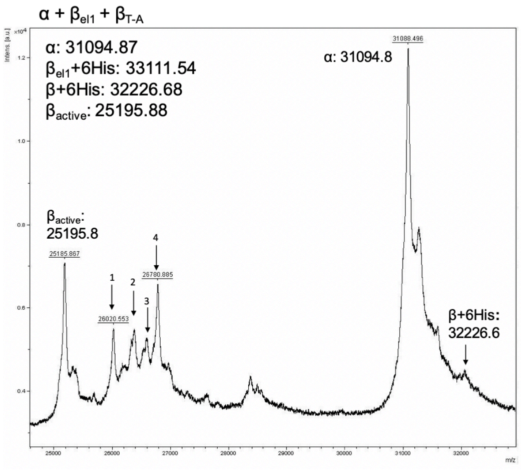

c

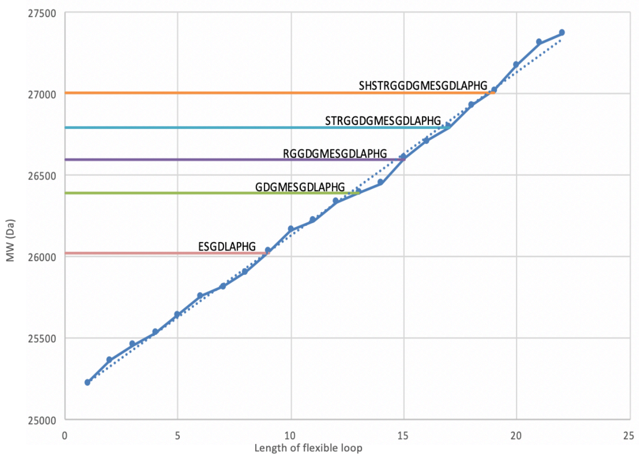

b

Peaks	Size of peaks from MALDI	Corresponding weight (Da) of beta polypeptide	Sequence of propeptide flexible loop	Difference in weight from next amino acid
β_{active}	25195.88	25165.86	Completely processed	30.02
1	26020	26029.76	ESGDLAPHG...	9.76
2	26385	26390.14	GDGMESGDLAPHG...	5.14
3	26595	26603.38	RGGDGMESGDLAPHG...	8.38
4	26788	26791.57	STRGGDGMESGDLAPHG...	3.57
* Peak exclusive for β_{sl1}	27006	27015.79	SHSTRGGDGMESGDLAPHG...	9.79

Figure S4. Analysis of the partially processed band formed during CP activation, Related to Figure 5.

- **a.** To estimate the size of partially processed band (Fig 4d), we performed MALDI analysis of the reconstitution mixture containing 4 μM of α, 2 μM of β_{el1} and 2 μM of $\beta_{TtoA.}$ Three out of four numbered peaks were also obtained upon analysis of reconstitution mixture of α with β_{sl1} and $\beta_{\Delta 23\ TtoA}$ (data not shown).
- **b.** Analysis of masses identified in (a). Comparison of the molecular weight of the peaks obtained by MALDI with the predicted molecular weight of β polypeptide truncated at different amino acid residues as shown in (c) where molecular weight v/s the length of flexible loop was plotted (counted backwards from -1 position). Peaks unique to the reconstitution mixture consisting of either β_{el1} or β_{sl1} are indicated in the table.

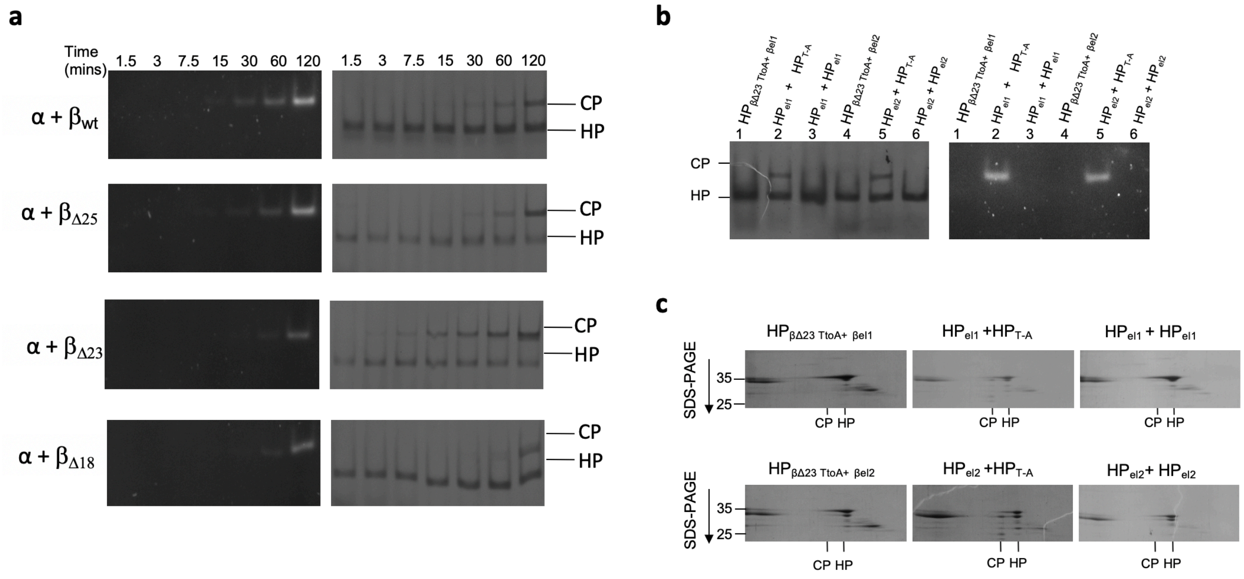

Figure S5. Delay in propeptides autocatalysis for N-terminal β truncations is also observed when preassembled HPs are allowed to dimerize at 30 °C, Related to Figure 7.

a. Equimolar amounts of α with indicated labelled Region I truncations were reconstituted at 4 °C overnight to allow for the formation of HPs. Next, HPs were reconstituted in 1:1 ratio at 30 °C and analyzed at indicated time points using peptidase activity assay and CBB staining following native-PAGE separation.
b. Similar to (a), dimerization using HPs formed by Region III mutants β_{el1} and β_{el2} was analyzed on native-PAGE
c. 2D-PAGE analysis of indicated lanes from (b) was done as described before.

Supplemental table 1. plasmids and primers used in thus study, Related to Figure 1 and 2.

Plasmid	Template	Primers	Protein expressed	Abbreviation
pJR659	pET22B	FP (5' GCGATGTATACATATGACCGCGGATCGTCCG 3')<br>RP (5'AAAATATATA CTCGAGACGCGCGCTGCCGCC 3')	N-(PrcB1 ORF)-6xHis-C	β_{wt}
pJR662	pTBSG	FP (5'-TACTTCCAATCCAATGCGATGACCATGCCGTACTACG-3')<br>RP (5'-TTATCCACTTCCAATGTTAGTCCTGAGTGTCGGCGG-3')	pTBSG-PrcA	α
pJR681	pJR659	FP: pRL437 (5'-GCCGCACGGCgcgACCATTGTGGCGCTGAC-3')<br>RP: pRL438 (5'-GCCAGGTCACCGCTCTCCATG-3')	N-(PrcB1$_{T\text{-}A}$ ORF)-6xHis-C	$\beta_{T\text{-}A}$
pJR770	pJR659	FP: pRL519 (5'-gaaagcggtGACCTGGCGCCGCACGGC-3')<br>RP: pRL520 (5'-gccaccgccACCGATACGGTTTTCCGGCAGCAG-3')	N-(PrcB1$_{sl1}$ ORF)-6xHis-C	β_{sl1}
pJR771	pJR659	FP: pRL521 (5'-ggcggtGACCTGGCGCCGCACGGC-3')<br>RP: pRL522 (5'-accgccACCGATACGGTTTTCCGGCAGCAG-3')	N-(PrcB1$_{sl2}$ ORF)-6xHis-C	β_{sl2}
pJR772	pJR659	FP: pRL523 (5'-ggccgtggcggtgatGGCATGGAGAGCGGTGAC-3')<br>RP: pRL524 (5'-gtcacctccgcgaccATCGCCACCACGGGTGCT-3')	N-(PrcB1$_{el1}$ ORF)-6xHis-C	β_{el1}
pJR773	pJR659	FP: pRL525 (5'-cggtgatggacgcggtggagatGGCATGGAGAGCGGTGAC-3')<br>RP: pRL526 (5'-ccacggccgtcacctccgcgaccATCGCCACCACGGGTGCT-3')	N-(PrcB1$_{el2}$ ORF)-6xHis-C	β_{el2}
pJR775	pJR779	FP: pRL463 (5'-GCCGCACGGCACCACCATTGTGGCGCTGAC-3')<br>RP: pRL438 (5'- GCCAGGTCACCGCTCTCCATG -3')	N-(Δ18 PrcB1 ORF)-6xHis-C	$\beta_{\Delta 18}$
pJR776	pJR780	FP: pRL463 (5'-GCCGCACGGCACCACCATTGTGGCGCTGAC-3')<br>RP: pRL438 (5'- GCCAGGTCACCGCTCTCCATG -3')	N-(Δ23 PrcB1 ORF)-6xHis-C	$\beta_{\Delta 23}$
pJR777	pJR781	FP: pRL463 (5'-GCCGCACGGCACCACCATTGTGGCGCTGAC-3')<br>RP: pRL438 (5'- GCCAGGTCACCGCTCTCCATG -3')	N-(Δ25 PrcB1 ORF)-6xHis-C	$\beta_{\Delta 25}$
pJR779	pJR681	FP: pRL441 (5'- TTTGGCAGCAATCTGAGCAGC-3')<br>RP: pRL449 (5'-CATATGTATATCTCCTTCTTAAAGTTAAACAAAATTATTTCTAGAGGG-3')	N-(Δ18 PrcB1 $_{TtoA}$ ORF)-6xHis-C	$\beta_{\Delta 18\ T\text{-}A}$
pJR780	pJR681	FP: pRL442 (5'- AGCAGCTTTACCGACTACC-3')<br>RP: pRL449 (5'-CATATGTATATCTCCTTCTTAAAGTTAAACAAAATTATTTCTAGAGGG-3')	N-(Δ23 PrcB1 $_{TtoA}$ ORF)-6xHis-C	$\beta_{\Delta 23\ T\text{-}A}$
pJR781	pJR681	FP: pRL443 (5'- TTTACCGACTACCTGCGTGGTC-3')<br>RP: pRL449 (5'-CATATGTATATCTCCTTCTTAAAGTTAAACAAAATTATTTCTAGAGGG-3')	N-(Δ25 PrcB1 $_{TtoA}$ ORF)-6xHis-C	$\beta_{\Delta 25\ T\text{-}A}$

Transparent Methods

Plasmids: *Rhodococcus erythropolis* PrcA gene was amplified using pT7-7 $\alpha 1$ (a generous gift from Wolfgang Baumeister) as template. The PCR amplified fragment was cloned in pTBSG plasmid (a generous gift from Philip Gao, Protein Production Group, University of Kansas) using ligation independent cloning to generate pTBSG$\alpha 1$, (pJR662). PrcB gene was similarly amplified from pT7-7$\beta 1$ plasmid (a generous gift from Wolfgang Baumeister). The PCR amplified fragment was cloned in Nde1 and Xho1 digested pET22B plasmid (a gift from Roberto DeGuzman, University of Kansas) resulting in generation of pET22B $\beta 1$, (pJR659). Plasmids used in this paper are listed in Supplementary Table 1. The accuracy of the plasmids generated was confirmed by sanger sequencing.

Protein expression and purification: For expression of recombinant proteins, a plasmid carrying the gene of interest was transformed into competent Rossetta cells (Novagen, BL-21 DE3 derived strain). The transformed cells were inoculated in 3 ml Luria-Bertani (LB) broth supplemented with 100 µg/mL ampicillin and 34 µg/ml chloramphenicol and incubated under shaking conditions at 37 °C overnight. This culture was used to inoculate 100 ml of the same media. At OD_{600} of 0.6 IPTG was added to a final concentration of 0.1 mM IPTG and the culture was incubated at 30 °C for 3 hours under continuous shaking. Cells were collected by centrifugation and pellets were stored at -80 °C. For purification of the His-tagged proteins, the pellet was re-suspended in lysis buffer (5 mM Imidazole, 20 mM Tris-HCl [pH8], 100 mM NaCl, 0.75 mM EDTA) supplemented with protease inhibitors (Roche proteasome inhibitor cocktail) and cells were then lysed by French Press at 900 psi. The total lysate was cleared by centrifugation (SS34 rotor, 10,000 rpm, 20 minutes, 4 °C). The cleared lysate was then incubated with 100 µl Roche complete His-Tag purification resin for 1 hour at 4 °C under rotation. The resin was collected be centrifugation (500 g, 4 minutes) and re-suspended in 5 ml wash buffer (15 mM Imidazole in PBS buffer (0.1 % Triton X-100, 0.02 % Sodium Azide)). The wash step was repeated 3 times. The His-tagged proteins were eluted step wise by resuspending the resin in 150 µl of elution buffer (50 mM Tris-HCl [pH7.5], 100 mM NaCl, 1 mM EDTA, 0.25 mM DTT) with increasing concentrations of Imidazole (60 mM imidazole, 120mM, 250mM, 400mM and 500mM). Fractions containing the protein of interest were pooled and dialyzed against dialysis buffer (50 mM Tris-HCl [pH7.5], 100 mM NaCl,1 mM EDTA, 0.25 mM DTT).

In vitro reconstitution assays: The reconstitution of the purified proteins and specified concentrations was done in the buffer containing 1 M Tris-HCl [pH7.5], 1 M $MgCl_2$, 0.5 mM ATP. The total reaction volume for assays was 25 µl. Reconstitutions were done at

30 °C unless stated otherwise. The reconstitution of subunits to form HPs was done at 4 °C.

Native gels and two dimensional (2D) assays: To analyze reconstituted samples by Native-PAGE , native gel loading buffer was added to samples (50 mM Tris-HCl [pH 7.4], 50% glycerol, 60 ng/ml 1-xylene cyanol). Samples were separated on a 3.6% native gel and analyzed for the activity using in-gel using the substrate suc-LLVY-AMC as described previously (Elsasser et al., 2005; Roelofs et al., 2018). Next, gels were then stained with Coomassie Brilliant Blue. For 2D-PAGE analysis, after an initial native-PAGE, the lane containing the sample of interest was excised from gel, incubated with 1X SDS-Sample buffer for 10 minutes, and loaded on a second dimension to separate the proteins based on their size using SDS-PAGE (Roelofs et al., 2018). After electrophoresis the gels were stained using Coomassie Brilliant Blue.

MALDI-ToF analyses: Matrix assisted laser desorption ionization-time of flight mass spectrometry (MALDI-TOF MS) on a Bruker Ultraflex II machine was used to determine masses of proteins. The protein solution was spotted with an equal amount of sinapic acid (Sigma-Aldrich, St. Louis, MO) matrix solution, and the samples were run in linear mode to determine protein mass.

Multiple sequence alignment of the bacterial β propeptide sequences: The β subunit of *Rhodococcus erythropolis* was used as an input query sequence to search the non-redundant NCBI protein sequence database for all bacterial species using the default parameters of BLASTp (Altschul et al., 1990; Sievers et al., 2011). The 256 sequences used for the multiple sequence alignment were selected based on sequence identity (more than 60% identity), best E-values, and manually curation (to avoid duplicates, incorrectly annotated sequences and hypothetical proteins). Next, to identify regions of similarity we performed a multiple sequence alignment (MSA) using the Clustal Omega package with the default parameters (Sievers et al., 2011). The resulting alignment, together with structural data from *R.e.* (PDB:1Q5R) and *Mtb* (PDB ID : 3MKA), allowed us to define three distinct regions in the propeptides. For *R.e.* Prcβ1 these were: i) Region I: From -65th to -43rd N terminal residues. ii) Region II : From -42nd to -27th residues. iii) Region III : From -26th to -4th residues (refer to the table in the Supplementary Material for more detail on these three regions). Calculation of average glycine content was performed by collecting all sequences from a given region (e.g. Region III) across all species, counting the number of glycine residues, and dividing by the total number of residues in that region across all species. In Region III, very few species deviated from the general observation of glycine enrichment (see Supplementary Material). Note that the conserved "PHG" motif at the C-terminal end of Region III was excluded from this analysis, since this final G residue is likely involved in promoting cleavage and thus would not be relevant to understanding the evolution of flexibility in Region III. Statistical

significance was calculated using a hypergeometric test, with the null hypothesis that the observed number of glycines in Region III would be obtained by placing residues at random in Regions I, II and III.

Molecular modelling and simulations: The systems were generated using the CHARMM-GUI (Jo et al., 2008) input generator by using the initial coordinates form the crystal structure of the mutant *R.e.* CP that is catalytically dead and thus retains the propeptide (PDB ID :1Q5R). HP models were generated by only selecting a HP from the starting CP structure. The missing electron density residues (residue IDs from -65 to -50 and -24 to -7) were modeled using the Galaxy-Fill tool as implemented directly in CHARMM-GUI (Coutsias et al., 2004). The proteins were solvated with water molecules in a periodic water box with 10 Å buffering distance between the protein surface and the box, using the TIP3P explicit water model (Jorgensen, 1983). Counterions of 0.1 M NaCl were added to neutralize the system.

The NAMD 2 (Phillips et al., 2005) program with the CHARMM C36m (Huang et al., 2017) force field was used to initiate all-atom Molecular dynamics simulations of the HP structure. Systems were equilibrated for 100ps using NVT (constant particle number, volume, and temperature) dynamics at 303.3 K without any restraints. The simulation systems measured about 143 × 143 × 143 Å^3 with total ~280,000 atoms. For production runs we used NPT (constant particle number , pressure, and temperature) dynamics with temperature and pressure held at 303.3 K and 1 bar, respectively. In the production runs, three replicates were simulated for 100ns using a local cluster and one replicate was run on the Anton 2 machine for 2 μs (Shaw et al., 2009). For the 100 ns runs we used a 2 fs time step and trajectories were saved every 2 fs. All the equilibration and production runs were performed using the default values based on the CHARMM-GUI input scripts (Brooks et al., 2009; Jo et al., 2008; Lee et al., 2016). For the Anton2 production run, the NPT ensemble was used with pressure and temperature maintained at 1bar and 303.3 K respectively, and the time step was 2 fs. Trajectories were saved every 240 ps. All analysis of the resulting trajectories was performed using the CHARMM simulation package and VMD.

Root Mean Square fluctuations (RMSF) of the propeptide in MD simulations: RMSF is a metric that is used to measure the fluctuation in the position of an atom or group of atoms. To calculate it, we first aligned each frame the starting structure, in order to ignore motions that arise from translation or rotation of the entire structure during the simulation. After alignment, RMSF is calculated as the standard deviation in position about the mean position, where the mean is taken by averaging the position of the residue across all (aligned) frames. For every replicate the reported RMSF values are averaged for all the

backbone atoms of a given residue (C, O, N and Cα) to calculate the residue-based RMSF. This residue-based RMSF is then averaged across all seven β propeptides to generate the RMSF for a given replicate.

Supplementary section for the Multiple Sequence Alignment

Using a BLASTP search algorithm with *R.e.* β sequence as the input yielded about 1000 hits with at least 60% sequence identity. This cutoff was chosen to include the β sequence from *M. tuberculosis*. Results were then filtered to remove redundant sequences, sequencing errors or misannotated sequences. This resulted in 256 distinct and non-redundant propeptide sequences. These were used in a Multiple sequence Alignment (MSA), using Clustal Omega with default parameters, which led to the identification of the glycine-rich Region III. Since MSA does not provide statistical estimates (Pearson, 2013), we conducted a hypergeometric test. This statistical test allowed us assess the significance of glycine enrichment in Region III and obtain a p-value that would indicate the chance that the glycine enrichment is not due to evolutionary pressure but resulted from random chance. This p-value is computing using the following formula:

$$p - value = P(x \geq b) = \sum_{k=b}^{min(K,n)} \frac{\binom{K}{k}\binom{N-K}{n-k}}{\binom{N}{n}}$$

where *N* is the total number amino acids in 256 sequences (13108) and *n* represents the number of glycines in all three regions (1200). *K* represents the number of amino acids in Region III (4380) and *b* denotes the total number of glycines in Region III (810). This p-value represents the chance of observing glycine enrichment of 17.8% or more in Region III under a null hypothesis where the residues are assigned to Regions I, II and III completely at random. The calculated *p*-value was 3.94×10^{-142} indicating strong evidence against null hypothesis. Thus, its highly likely that glycine enrichment of Region III is caused by evolutionary pressure and highly unlikely that it occurred by chance.

Supplemental references

Altschul, S.F., Gish, W., Miller, W., Myers, E.W., and Lipman, D.J. (1990). Basic local alignment search tool. J Mol Biol *215*, 403-410.

Berman, H.M., Westbrook, J., Feng, Z., Gilliland, G., Bhat, T.N., Weissig, H., Shindyalov, I.N., and Bourne, P.E. (2000). The Protein Data Bank. Nucleic Acids Res *28*, 235-242.

Brooks, B.R., Brooks, C.L., 3rd, Mackerell, A.D., Jr., Nilsson, L., Petrella, R.J., Roux, B., Won, Y., Archontis, G., Bartels, C., Boresch, S.*, et al.* (2009). CHARMM: the biomolecular simulation program. J Comput Chem *30*, 1545-1614.

Coutsias, E.A., Seok, C., Jacobson, M.P., and Dill, K.A. (2004). A kinematic view of loop closure. J Comput Chem *25*, 510-528.

Elsasser, S., Schmidt, M., and Finley, D. (2005). Characterization of the Proteasome Using Native Gel Electrophoresis. In Ubiquitin and Protein Degradation, Part A, pp. 353-363.

Huang, J., Rauscher, S., Nawrocki, G., Ran, T., Feig, M., de Groot, B.L., Grubmuller, H., and MacKerell, A.D., Jr. (2017). CHARMM36m: an improved force field for folded and intrinsically disordered proteins. Nat Methods *14*, 71-73.

Jo, S., Kim, T., Iyer, V.G., and Im, W. (2008). CHARMM-GUI: A web-based graphical user interface for CHARMM. Journal of Computational Chemistry *29*, 1859-1865.

Jorgensen, W.L., Chandrasekhar, J., Madura, J.D. (1983). Comparison of simple potential functions for simulating liquid water. J. Chem. Phys. *79*, 926-935.

Kabsch, W., and Sander, C. (1983). Dictionary of protein secondary structure: pattern recognition of hydrogen-bonded and geometrical features. Biopolymers *22*, 2577-2637.

Lee, J., Cheng, X., Swails, J.M., Yeom, M.S., Eastman, P.K., Lemkul, J.A., Wei, S., Buckner, J., Jeong, J.C., Qi, Y.*, et al.* (2016). CHARMM-GUI Input Generator for NAMD, GROMACS, AMBER, OpenMM, and CHARMM/OpenMM Simulations Using the CHARMM36 Additive Force Field. J Chem Theory Comput *12*, 405-413.

Pearson, W.R. (2013). An introduction to sequence similarity ("homology") searching. Curr Protoc Bioinformatics *Chapter 3*, Unit3 1.

Phillips, J.C., Braun, R., Wang, W., Gumbart, J., Tajkhorshid, E., Villa, E., Chipot, C., Skeel, R.D., Kale, L., and Schulten, K. (2005). Scalable molecular dynamics with NAMD. J Comput Chem *26*, 1781-1802.

Roelofs, J., Suppahia, A., Waite, K.A., and Park, S. (2018). Native Gel Approaches in Studying Proteasome Assembly and Chaperones. Methods Mol Biol *1844*, 237-260.

Shaw, D.E., Dror, R.O., Salmon, J.K., Grossman, J., Mackenzie, K.M., Bank, J.A., Young, C., Deneroff, M.M., Batson, B., and Bowers, K.J. (2009). Millisecond-scale molecular dynamics simulations on Anton. In Proceedings of the conference on high performance computing networking, storage and analysis (ACM), p. 39.

Sievers, F., Wilm, A., Dineen, D., Gibson, T.J., Karplus, K., Li, W., Lopez, R., McWilliam, H., Remmert, M., Soding, J*., et al.* (2011). Fast, scalable generation of high-quality protein multiple sequence alignments using Clustal Omega. Mol Syst Biol *7*, 539.
